# A novel drug–drug nanohybrid for the self-delivery of porphyrin and *cis*-platinum†

**DOI:** 10.1039/c9ra07085k

**Published:** 2019-11-12

**Authors:** Changfu Shan, Jiaxi Ru, Meina Zhang, Jing Cao, Weisheng Liu, Huichen Guo, Yu Tang

**Affiliations:** State Key Laboratory of Applied Organic Chemistry, Key Laboratory of Nonferrous Metal Chemistry and Resources Utilization of Gansu Province, College of Chemistry and Chemical Engineering, Lanzhou University Lanzhou 730000 P. R. China tangyu@lzu.edu.cn caoj@lzu.edu.cn; State Key Laboratory of Veterinary Etiological Biology, Lanzhou Veterinary Research Institute, Chinese Academy of Agricultural Sciences Lanzhou 730046 P. R. China guohuichen@caas.cn

## Abstract

The thriving development of nanotechnology has greatly promoted the development of drug delivery systems (DDSs) in the past decades. However, most DDSs themselves cannot serve as diagnostic reagents and must be metabolized, by which they may become poisonous and even cause immune reactions. In this study, a novel self-delivery drug–drug system (SDDS) nanohybrid based on the coordination assembly of a photodynamic reagent, tetra-(4-carboxyphenyl)porphyrin (TCPP), and a chemotherapy reagent, *cis*-platinum, was designed and synthesized. The four carboxyl groups of TCPP can compete with the chloride ions of *cis*-platinum by coordination interactions, forming a TCPP-*cis*-platinum nanohybrid (PCNH) for the purpose of photodynamic/chemotherapeutic synergistic treatment with a combinational index of 0.28. Meanwhile, the PCNH system can effectively protect the photosensitizer TCPP from photobleaching when irradiated continuously in the photodynamic therapy (PDT) process, which is very crucial for PDT. Furthermore, introduction of the heavy atom platinum can greatly enhance the producing efficiency of ^1^O_2_ by 46%. In addition, the red emission fluorescence of TCPP is beneficial for monitoring and tracing the process of drug delivery when used *in vitro*. This work may pave a new way for the design of new integrated nanohybrids for diagnosis and synergistic treatment.

## Abstract 2

A novel self-delivery drug–drug system nanohybrid constructed by the coordination assembly of porphyrin and *cis*-platinum was used for synergetic photodynamic therapy and chemotherapy.

## Graphical abstract

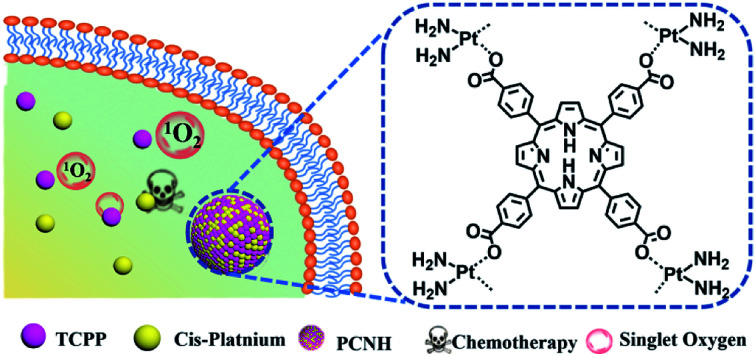

## 1 Introduction

Precise diagnosis and treatment are of great significance for highly selective and efficient cancer therapy. In traditional cancer treatments, doxorubicin (DOX), paclitaxel (PTX), camptothecin (CPT) and *cis*-platinum remain indispensable owing to their advantages of mature theories and application.^1–3^ However, these micromolecule drugs possess inherent limitations, including random distribution, poor solubility, low bioavailability and short cycling periods, which clinically result in dose-dependent toxicity.^4^ Benefiting from the active development of nanotechnology, nanomedicine can endow micromolecule drugs with better solubility, chemical stability and decreased biodegradation or excretion.^2,5,6^ Certain sizes of molecules, such as liposomes, nanoparticles, and macromolecular drugs, tend to accumulate in tumour tissue much more than they do in normal tissues; this is known as the enhanced permeability and retention effect (EPR).^7^ Numerous research results show that the EPR effect can enhance the accumulation of drugs in tumours up to 5 to 10 fold.^1^ Meanwhile, elaborately designed drug delivery systems (DDS) can also release their cargo spatio-temporally when triggered endogenously (pH, redox, enzymes) or exogenously (light, heat, magnetism, ultrasound).^8–11^ As a result, large amounts of drug carriers have been developed in the progress of nanomedicine, such as mesoporous silicon, magnetic nanoparticles, polymer materials, quantum dots, and gold and carbon nanomaterials.^12,13^

However, introducing non-drug materials as carriers causes two problems. One is limited loading efficiency because the carriers should occupy a certain mass.^14^ The other is safety issues. Some of the metabolites of carriers are poisonous and even cause immune reactions, which brings great risk to patients and impedes the clinical use of nanomedicines.^15^ To resolve these carrier-dependent problems, the self-delivery concept was proposed, where the carriers are constructed only from drugs.^16^ This concept tremendously improves the delivery efficiency and fundamentally decreases side effects by eliminating the use of non-drug materials as carriers. To the best of our knowledge, most self-delivery drug–drug systems (SDDS) are constructed based on covalent bonds or supramolecular interactions such as hydrogen bonding, π–π stacking, C–H⋯π interactions, van der Waals interactions and halogen bonding.^17^ As a member of the supramolecular interaction family, coordination interactions between different drugs have rarely been used to construct SDDS. Meanwhile, coordination bonds should be broken in weak acidic neoplastic microenvironments; thus, drugs can be released precisely within tumour cells by an endogenous trigger.

**Scheme 1:**
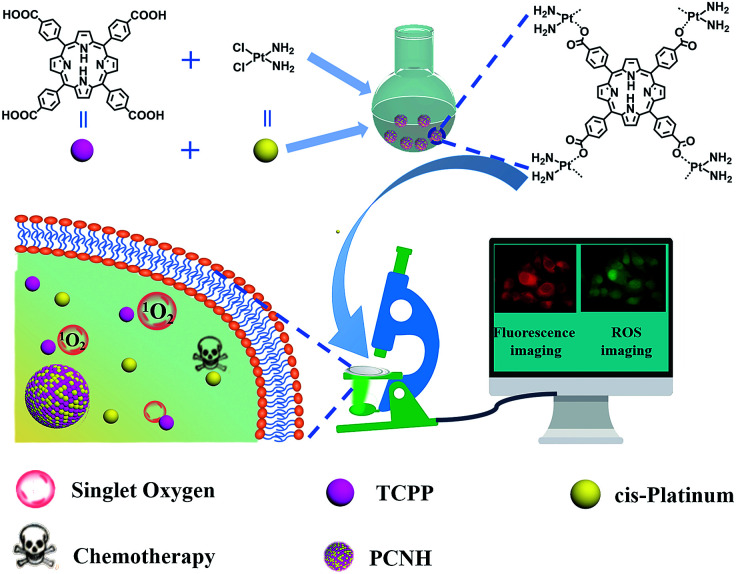
Illustration of the synthetic procedure of PCNH and the imaging-guided synergetic therapy of PDT and chemotherapy.

Herein, we designed a SDDS nanohybrid based on the coordination assembly of a photodynamic reagent, 5,10,15,20-tetra(4-carboxylphenyl)porphyrin (TCPP), and a chemotherapy reagent, *cis*-dichlorodiammine platinum(ii) (*cis*-platinum) (Scheme 1). The design strategy of the TCPP-*cis*-platinum nanohybrid (PCNH) was established based on coordination characteristics, where platinum can combine with the carboxyls of TCPP more strongly than chloride ions under weak alkaline conditions. Meanwhile, the four carboxyls of TCPP should be available to construct topological structures, and *cis*-platinum can function as a bridge to link TCPP together. In contrast to normal physiological pH (7.2 to 7.4), the extracellular pH around solid tumours is acidic (pH 6.5 to 6.9), and that in late endosomes is even more acidic (pH 5.5).^18^ The coordination bonds between –COO^−^ and Pt can be broken in the weakly acidic neoplastic microenvironment. Thus, TCPP and *cis*-platinum can be released from the nanohybrid PCNH, which is fundamental for synergistic therapy of photodynamic therapy (PDT) and chemotherapy. Meanwhile, due to the heavy metal effect of the platinum coordinated with TCPP, the production efficiency of singlet oxygen can be elevated by 46% over TCPP as a reference under the same conditions. Also, PCNH can effectively protect the photosensitizer from photobleaching when continuously irradiated by light, and the red fluorescence of TCPP can be used to monitor the process of drug delivery.

## 2 Experimental

### 2.1 Materials

All chemicals were used without purification unless otherwise specified. Methyl-4-formylbenzoate, *cis*-platinum and pyrrole were purchased from HEOWNS (Tianjin, China). 3-(4,5-dimethylthiazol-2-yl)-5-(3-carboxymethoxyphenyl)-2-(4-sulfophenyl)-2*H*-tetrazolium (MTS), HOTEST-33258 and 2′,7′-dichlorodihydrofluorescein diacetate (DCFH-DA) were purchased from YESEN (Shanghai, China). Fetal bovine serum (FBS) was purchased from Biosharp Life Science (Guangzhou, China). Cell culture medium was purchased from Hyclone Laboratories, GE Healthcare Life Science (Utah, America). Other reagents were all purchased from Rionlon (Tianjin, China).

### 2.2 Instruments

UV-vis absorption spectra were recorded on a UV-vis-NIR spectrometer (Agilent Technologies, Cary-5000, America); FT-IR spectra were recorded on an FT-IR spectrometer (Bruker, Vertex 70, Germany) with the KBr method; fluorescence spectra were recorded on a fluorescence spectrophotometer (Shimadzu, RF5301-PC, Japan; Edinburgh, FLS 920, Germany); near infrared (NIR) spectra were measured by an Edinburgh Instruments spectrometer (FLS 920, UK); ^1^H-NMR spectra were recorded on a JNM-ECS 400 MHz spectrometer with TMS as the internal reference; transmission electron microscopy (TEM) images and EDX were recorded on a TECNAI G2 with 200 kV accelerating voltage (TECNAI G2, America); cell images were recorded on a fluorescent microscope with blue light as the excitation (Olympus, IX71, Japan) or on a laser scanning confocal microscope (Leica, SP8, Germany). The *ζ*-potential was recorded on a Malvern Instruments system (UK).

### 2.3 Synthesis of 5,10,15,20-tetra(4-methoxycarbonylphenyl)porphyrin (TCPP-OMe)

TCPP-OMe was synthesized according to the literature with slight modification.^19^ Methyl-4-formylbenzoate (16.5 g, 100 mmol) was dissolved in *n*-propionic acid (250 mL), and the solution was heated to 135 °C until slightly boiling. Newly distilled pyrrole (6.7 mL) in *n*-propionic acid (30 mL) was dropped into the solution within 20 min, and the colour became dark. After 1 h, the temperature was allowed to cool to 80 °C, and some of the *n*-propionic acid (100 mL) was removed by a rotary evaporator. The mixture was stored in a refrigerator (4 °C) overnight and the sediment was filtered, followed by washing with water and ethanol three times. The crude product was purified by column chromatography with CH_2_Cl_2_ as the eluent. ^1^H-NMR (400 MHz, DMSO-d6): *δ* 8.81 (s, 8H), 8.44 (d, 8H, *J* = 8.4 Hz), 8.29 (d, 8H, *J* = 8.4 Hz), 4.10 (s, 12H), −2.83 (s, 2H). Micro-TOF, *m*/*z* calcd [M + H]^+^: 846.27, found: 846.40.

### 2.4 Synthesis of 5,10,15,20-tetra(4-carboxylphenyl)porphyrin (TCPP)

TCPP was synthesized by hydrolysing TCPP-OMe. Briefly, TCPP-OMe (500 mg) was transferred into a mixed solution containing methanol (100 mL), THF (50 mL) and 40% KOH (12 mL). After the mixture was heated at 40 °C for 4 h, the temperature was allowed to cool to R.T. and the pH was adjusted to 4 to 5 by adding concentrated hydrochloric acid in an ice-water bath. Next, the sediment was removed by filtration, and the TCPP was obtained by removing the solvent using a rotary evaporator. Lastly, the crude product was washed with large amounts of water and CH_2_Cl_2_. ^1^H-NMR (400 MHz, DMSO-d_6_): *δ* 13.0 (S, COOH), 8.38 (d, 8H, *J* = 8.0 Hz), 8.32 (d, 8H, *J* = 8.0 Hz), −2.86 (s, 2H).Micro-TOF, *m*/*z* calcd [M + H]^+^*m*/*z* = 791.21, found *m*/*z* = 791.18.

### 2.5 Synthesis of porphyrin *cis*-platinum nanohybrid (PCNH)

*cis*-Platinum (15.0 mg) in DMF (1 mL) was dropped into DMF (25 mL) which contained TCPP (19.8 mg) within 10 min. When the temperature was elevated to 120 °C, triethylamine (80 μL, 120 μL, 160 μL, 320 μL and 640 μL, respectively) was dropped within 2 days; then, the temperature was allowed to cool to room temperature. Meanwhile, to study the assembly behaviour in water, TCPP was dissolved in water and the pH was adjusted to 8. Then, *cis*-platinum in 5 mL water was added dropwise, and the temperature was elevated to 50 °C for 8 h. PCNH was obtained by centrifugation (18 000 r, 20 min) and washed with DMF and water. Finally, the PCNH was dried with freeze drying equipment.

### 2.6 Determination of fluorescence quantum yields

TCPP and PCNH were dissolved or distributed in D_2_O, and the pH value was adjusted to 7.24 with triethylamine. The absorbance was controlled below 0.05 under the special peaks. The fluorescence data were subsequently collected. Fluorescence quantum yields were calculated according to the formula below:*Φ*_1_/*Φ*_2_ = (*A*_2_×*F*_1_)/(*A*_1_×*F*_2_)where *Φ* refers to the fluorescence quantum yield, *A* refers to the absorbance value and *F* refers to the integral area of the fluorescence spectrum.

### 2.7 Calculation of combination index (CI)

The combination index is an important parameter to distinguish different drugs that have synergistic, antagonistic or additive effects when used simultaneously. CI was calculated using the following formula:^20^CI = *D*_1_/*D*_f1_ + *D*_2_/*D*_f2_ + *D*_1_*D*_2_/*D*_f1_*D*_f2_where *D*_f1_ is the dose of TCPP alone required to achieve *X* percent viability and *D*_1_ is the dose of TCPP in PCNH required to achieve the same cell viability. Similarly, *D*_f2_ is the dose of *cis*-platinum alone required to achieve *X* percent viability and *D*_2_ is the dose of *cis*-platinum in PCNH required to achieve the same cell viability. In this paper, *X* equals 50%, that is, the IC_50_ value.

### 2.8 Test of cytotoxicity *in vitro*

The cytotoxicity was tested on HeLa cells using the MTS method.^21^ In short, HeLa cells were cultured in 96 well plates at a density of 5000 cells per well with 10% FBS cell culture medium at 37 °C in a 5% CO_2_ atmosphere. The experiment was conducted in the dark first. PCNH, TCPP and *cis*-platinum were added respectively, and the concentrations were 2.5 μg mL^−1^, 5.0 μg mL^−1^, 10.0 μg mL^−1^, 20.0 μg mL^−1^, and 40.0 μg mL^−1^, respectively (the concentration corresponds to pure TCPP and TCPP in PCNH); 24 h later, 10 μL MTS was added to each well. After 3 h, the optical density (OD) at 490 nm was recorded. Meanwhile, to verify the effects of photodynamic therapy, the experimental procedures were repeated, except that the 96-well plates were irradiated by an LED lamp (the total power was 20 W and the time was 10 min). The cell viability was calculated according to the formula below:Cell viability (%) = (OD_drug_/OD_control_)×100%where OD_drug_ refers to the OD value with the addition of TCPP, *cis*-platinum or PCNH and OD_control_ refers to the OD value of a well without addition of TCPP, *cis*-platinum or PCNH.

### 2.9 Test of singlet oxygen *in vitro*

The singlet oxygen *in vitro* was tested in a Petri dish. In brief, HeLa cells were cultured for 3 h. Then, 1 μL DCFH-DA was added, and the culture was continued for another 20 min. The fluorescence images were recorded on a confocal laser scanning microscope or fluorescence microscope with green light excitation. To record the increasing fluorescence intensity of singlet oxygen, photos were taken every 2 min with blue light as excitation.

## 3 Results and discussion

**Fig. 1:**
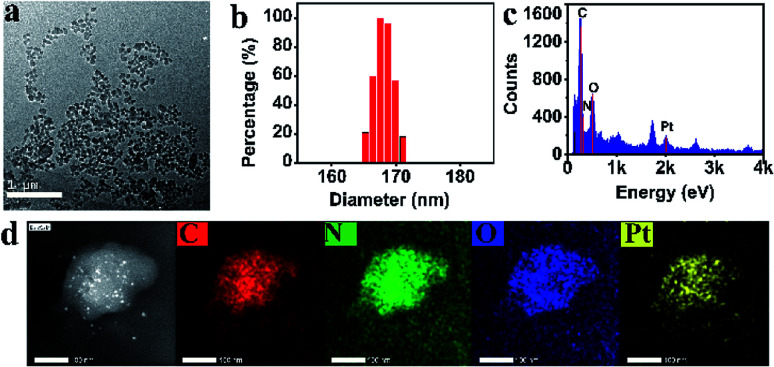
TEM ((a), scale bar 1 μm), DLS (b), EDX (c), and STEM results and EDS elemental mapping of PCNH ((d), scale bar 100 nm).

As mentioned above, PCNH was synthesized based on competitive coordination of the carboxyls (–COOH) of TCPP against the chloride ions of *cis*-platinum. Because the coordination ability of carboxyls is greatly influenced by the deprotonation degree, the shape of PCNH varied from rods with micron-sized lengths to spheres with 117 nm in diameter when the amount of triethylamine was varied. However, when a larger amount of triethylamine was added, the nanoparticles become crosslinked (Fig. S3, ESI†). On the other hand, when the assembly proceeded in water, the morphology became severely crosslinked. All the mentions of PCNH below refer to the 117 nm nanospheres from the transmission electron microscope image (Fig. 1a). The average hydrodynamic diameter of PCNH characterized by dynamic light scattering (DLS) is 168 nm (Fig. 1b); this is slightly larger than the value obtained by TEM, which can be ascribed to the dry state in TEM and the hydrodynamic diameter in DLS.^22^ Meanwhile, the energy-dispersive X-ray (EDX) (Fig. 1c) and energy dispersive spectrometer (EDS) elemental mapping data (Fig. 1d) clearly demonstrate that carbon, nitrogen, oxygen and platinum were successfully integrated into PCNH.

**Fig. 2:**
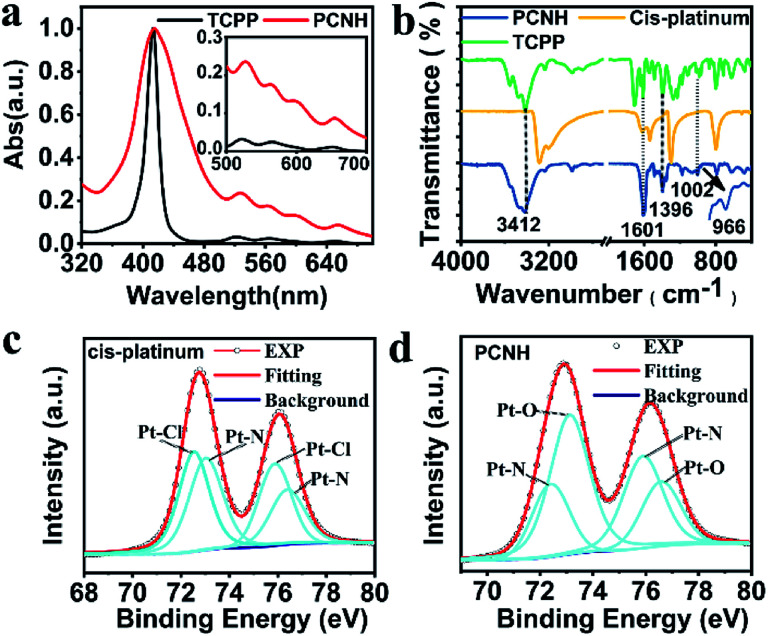
Normalized UV-vis spectra of TCPP and PCNH ((a); the insert is the amplification figure of the Q bands). FT-IR spectra of TCPP, *cis*-platinum and PCNH (b). XPS fine spectra of *cis*-platinum (c) and PCNH (d).

Fig. 2a shows the normalized UV-vis absorption of TCPP and PCNH. The characteristic absorption peak of TCPP, which belongs to the Soret band, was located at 419 nm. Meanwhile, the remaining four peaks at 515 nm, 550 nm, 589 nm and 646 nm are the peaks of the Q bands. Compared with TCPP, the peaks of the Q bands in PCNH are bathochromically shifted, while the Soret band in PCNH stayed the same; this is different from a pyrrole ring-metal complex, in which case the Soret band moved obviously.^23^ Importantly, as shown in Scheme 1, the combination of TCPP and *cis*-platinum was achieved *via* the coordination of platinum with carboxyl groups rather than the pyrrole ring. As a result, the symmetry of the pyrrole ring will not be influenced; thus, the energy levels will not be degenerate. Gratifyingly, the number of Q bands did not decrease, which implies that the symmetry indeed does not change and that no degeneracy of energy levels occurred. From these, we inferred that the platinum ions were coordinated with the carboxyls in the nanohybrid.^24^ As such, the molar ratio of [TCPP^4−^] and [Pt(NH_2_)_2_]^2+^ in PCNH is 1 : 2, which implies that the theoretical mass percent of platinum is 31.9%. ICP-MS gave a result of 30.5% for the mass percent of platinum in PCNH, which is consistent with the theoretical value. Meanwhile, the surface potential is an important parameter for drug delivery carriers because it greatly influences the mobility and cycling time when used *in vivo*. In comparison with TCPP, the *ξ* potential of PCNH increased because of the positive value of *cis*-platinum (Table S1, ESI†). Simultaneously, a slight negative potential is beneficial for PCNH to circulate in blood vessels.^25^

Fourier transform infrared (FT-IR) spectra were also used to verify the combination mode of TCPP with *cis*-platinum (Fig. 1d). After assembling with TCPP to form PCNH, the stretching vibration peak at 1704 cm^−1^ from the C=O of –COOH was bathochromically shifted. The peaks located at 1601 cm^−1^, 1534 cm^−1^, 1396 cm^−1^, and 1360 cm^−1^ belong to *ν*_as_(COO^−^) and *ν*_s_(COO^−^). The band gap between 1601 cm^−1^ and 1396 cm^−1^ is 205 nm, which implies that the carboxyl is a monodentate ligand in this situation.^26,27^ The characteristic peak of the pyrrole ring N–H rocking vibration at 966 cm^−1^ was still present, indicating that the platinum ions mainly coordinate with the carboxyls rather than the porphyrin rings.

X-ray photoelectron spectroscopy (XPS), a highly surface-sensitive probe, was also used to ascertain the success of the design strategy. Binding energies centred at *ca.* 75.7 eV, 199.5 eV, 284.8 eV, 401.6 eV and 533.1 eV were assigned to Pt 4f, Cl 2p, C 1s, N 1s and O 1s, respectively. In contrast with *cis*-platinum, the peak of Cl 2p disappeared completely, which intuitively shows that the chloride ions were removed in PCNH (Fig. S4, ESI†). The platinum in PCNH functioned as a bridge to link TCPP together by coordination. To further analyse the coordination environment of the platinum ions, the fine spectrum of Pt 4f was examined. The binding energies located at 75.75 eV and 72.49 eV belong to Pt 4f_5/2_ and Pt 4f_7/2_ of pure *cis*-platinum (Fig. 2c). However, both binding energies increased when *cis*-platinum coordinated with TCPP to construct PCNH (Fig. 2d), which can be explained by the fact that the coordination bond with platinum became stronger. Importantly, the binding energies of the Pt–N bond at 76.61 eV and 73.13 eV were maintained.^28^ However, the peaks at 75.75 eV and 72.41 eV which belong to the Pt–Cl binding energy vanished in PCNH.^29^ Meanwhile, two peaks at 75.87 eV and 72.43 eV appeared, which can be assigned to the Pt–O bond. The XPS data provide strong evidence that PCNH was constructed by coordination bonding between the carboxyls and platinum ions (Fig. 2d).

**Fig. 3:**
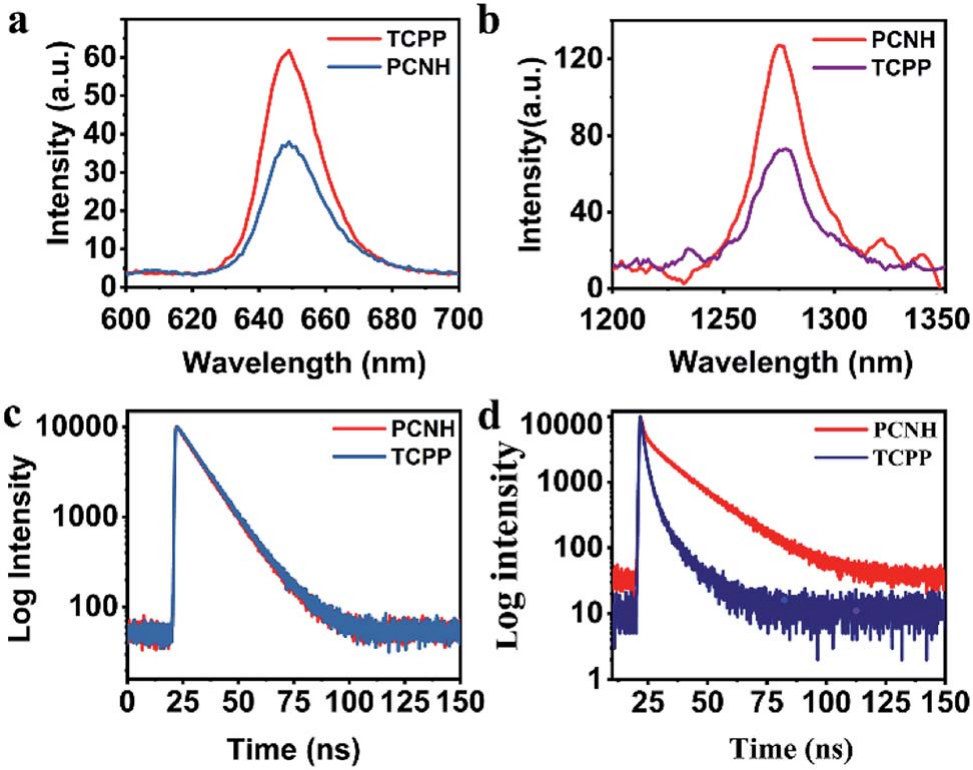
Fluorescence spectra (a) and singlet oxygen fluorescence spectra of TCPP and PCNH (b). Fluorescence lifetime spectra of TCPP and PCNH at room temperature (c) and liquid nitrogen temperature (d). All the excited wavelengths are 515 nm.

**Fig. 4:**
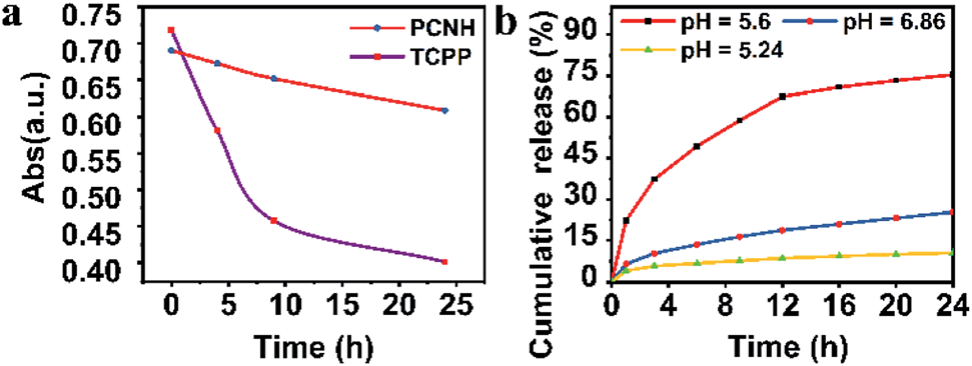
Photostabilities of PCNH and TCPP when exposed to natural light for 24 h (a). Cumulative release of TCPP from PCNH in different pH solutions ((b); the concentration of PCNH is 1 mg mL^−1^).

Benefiting from the long wavelength fluorescence emission of porphyrin, PCNH can be used as a good imaging reagent for tracing and diagnosis. The emission peaks for both TCPP and PCNH were centred around 650 nm (Fig. 3a). Although the fluorescence quantum yield of PCNH (*Q*_p_) decreased to 67.8% with TCPP as a reference under the same conditions (PBS buffer, pH 7.24), the value of *Q*_p_ was still acceptable and strong enough for cell imaging.^30^ Moreover, the wavelength of fluorescence was situated at the edge of the near-infrared I imaging window, which can help eliminate interference from bioluminescence and decrease the absorption from surrounding tissues.^31^ Additionally, when excited both at the Soret band and Q bands for both TCPP and PCNH, a singlet oxygen fluorescence emission peak at 1276 nm will appear (Fig. S5, ESI†). Because the lifetime of singlet oxygen is shorter than 200 ns and the diffusion distance is no longer than 20 nm,^32^ ensuring appropriate singlet oxygen quantum yields is very crucial for PDT. When excited by 515 nm light, although the absorbance intensity was similar (Fig. S6, ESI†), the singlet oxygen fluorescence intensity of PCNH was obviously stronger than that of TCPP (Fig. 3b) and the singlet oxygen quantum yield of PCNH was 46% higher than that of TCPP under the same conditions; this is due to the heavy atom effect of platinum and the prolonged lifetime of the triplet state of the photosensitizer (Fig. 3c and d).^33^ Furthermore, the photostability was also characterized by UV-vis spectra after exposure to natural light for 24 h. The absorbance of TCPP decreased by 43.9%, while that of PCNH decreased by only 11.8%; this implies that the photosensitizers can be effectively protected when integrated into the nanohybrid (Fig. 4a and S7 in the ESI†). The enhancement of photostability is very crucial for practical applications because photostability is a prerequisite for continuously producing toxic singlet oxygen.

As mentioned above, the formation of PCNH was based on the coordination of TCPP^4−^ with *cis*-platinum. However, the coordination ability of TCPP^4−^ relies on the pH value. In an acid environment, PCNH will disassemble gradually. The percentage of accumulatively released TCPP was quantified (Fig. 4b). Only 10% of TCPP was released when the pH value was 7.24. However, when the pH value decreased to 5.02, 74% of the TCPP disassembled from PCNH within 24 h. These results showed that PCNH is relatively stable in a normal physiological environment and can slowly disassemble in the acidic tumor microenvironment.

**Fig. 5:**
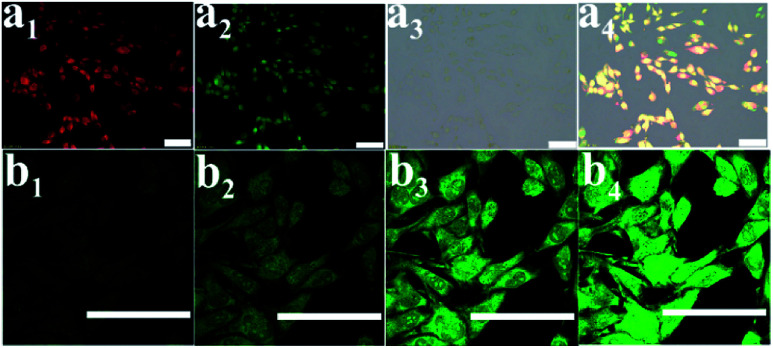
Fluorescence images of HeLa cells (a) red channel indicating PCNH fluorescence (a_1_), green channel corresponding to DCFH-DA (a_2_), bright field (a_3_) and the merged image (a_4_). (b) ROS cumulative production photographs taken by a confocal laser scanning microscope with DCFH-DA as the indicator (excitation interval is 2 min).

Considering that the PCNH nanohybrid contained a porphyrin emission centre, it can help monitor the procedure of drug delivery. Fluorescence imaging of PCNH was investigated *in vitro* on HeLa cells at a concentration of 20 μM. After cultivation for 3 h, the whole cells were lit with red fluorescence; this indicates that PCNH can be taken up by HeLa cells, which is beneficial for photodynamic therapy (Fig. 5a_1_). Furthermore, DCFH-DA was used as a ROS sensor (Fig. 5a_2_). The green fluorescence of DCFH-DA coincided well with the red fluorescence of TCPP, which further shows that PCNH can act not only as a photosensitizer but as an imaging reagent to monitor the procedure of drug delivery (Fig. 5a_4_). With increasing irradiation time, the singlet oxygen accumulated ceaselessly, and the green fluorescence became stronger (Fig. 5b).

**Fig. 6:**
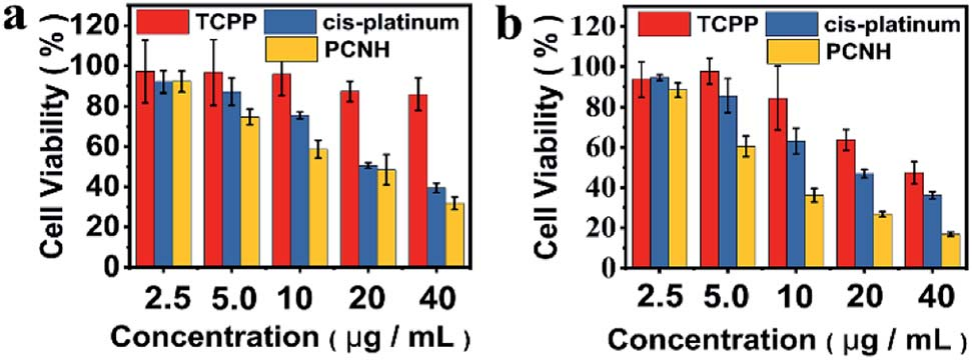
Cell viability of HeLa cells after incubation with different concentrations of TCPP, *cis*-platinum and PCNC in the dark (a) or with LED ((b), total power: 20 W).

To verify the therapeutic effects *in vitro*, the MTS assay was used on HeLa cells with an LED lamp as the irradiation source. Because *cis*-platinum kills cancer cells by binding and twisting DNA,^34^ light has negligible influence on cell viability when only *cis*-platinum is added (Fig. 6a and b). Because light is one of the prerequisites for PDT, the cell viability greatly relies on light irradiation when TCPP is added. Therefore, the viability remains at 85% in the dark when 40 μM TCPP is added (Fig. 6a); however, this value decreased to 47% at the same concentration when the cells were irradiated (Fig. 6b). Meanwhile, the IC_50_ values for TCPP, *cis*-platinum, and PCNH were 35.4 ± 1.2 μg mL^−1^, 17.2 ± 1.1 μg mL^−1^ and 6.8 ± 1.1 μg mL^−1^ with light (Fig. S8, ESI†), respectively. The combination index obtained by IC_50_ was much smaller than 1 (combination index = 0.28), which shows the great synergistic effect of chemotherapy and PDT for PCNH; this may benefit the different mechanisms of treatments.^20,35^

## 4 Conclusions

In conclusion, a novel SDDS nanohybrid named PCNH, based on the coordination assembly of TCPP and *cis*-platinum, was designed and synthesized. Because the nanohybrid PCNH only contains two drugs, high loading capacity can be easily realized. Meanwhile, PCNH can effectively protect TCPP from photobleaching, which is very crucial for PDT. Due to the heavy effect of platinum ions, which were coordinated with TCPP, the singlet oxygen quantum yield increased by 46%. Furthermore, on the basis of the coordination assembly of the photosensitizer TCPP, which is widely used in PDT, and *cis*-platinum, which is used in chemotherapy, synergetic therapy with high efficiency can be realized; the combination index is only 0.28. This work may pave a new way for the design and progress of SDDs and precise cancer treatment.

## Conflicts of interest

There are no conflicts to declare.

## Supplementary Material

RA-009-C9RA07085K-s001
