# Supplementary material for: A novel drug–drug nanohybrid for the self-delivery of porphyrin and cis-platinum
Source: RSC Adv. 2019 Nov 12;9(63):37003–8. doi: 10.1039/c9ra07085k (PMC9075146; doi:10.1039/c9ra07085k)
Supplement: RA-009-C9RA07085K-s001 [file RA-009-C9RA07085K-s001.pdf]

Electronic Supplementary Material (ESI) for RSC Advances.
This journal is © The Royal Society of Chemistry 2019

Electronic Supporting Information (ESI)

A Novel Drug-drug Nanohybrid for Self-delivery of Porphyrin and Cis-platinum†

Changfu Shan,[a] Jiaxi Ru,[b] Meina Zhang,[a] Jing Cao,*[a] Huicheng Guo,*[b] and Yu Tang*[a]

[a] State Key Laboratory of Applied Organic Chemistry, Key Laboratory of Nonferrous Metal Chemistry and Resources Utilization of Gansu Province, College of Chemistry and Chemical Engineering, Lanzhou University, Lanzhou, 730000, PR China

[b] State Key Laboratory of Veterinary Etiological Biology, Lanzhou Veterinary Research Institute, Chinese Academy of Agricultural Sciences, Lanzhou, Gansu Province 730046, P. R. China

Contents

1. **Experimental procedures**

   1.1 Synthetic process

   1.2 ^{1}H NMR spectrum

2. **Results and discussion**

   2.1 TEM images of PCNH

   2.2 ξ potential of the samples

   2.3 X-Ray photoelectron spectroscopy (XPS)

   2.4 Fluorescence spectra of singlet oxygen

   2.5 UV-vis absorbance spectrum of TCPP and PCNH

   2.6 Different photostability between TCPP and PCNH

   2.7 Cell viability against different concentration of TCPP, cis-platinum and PCNH

1. Experimental procedures

1.1 Synthetic process

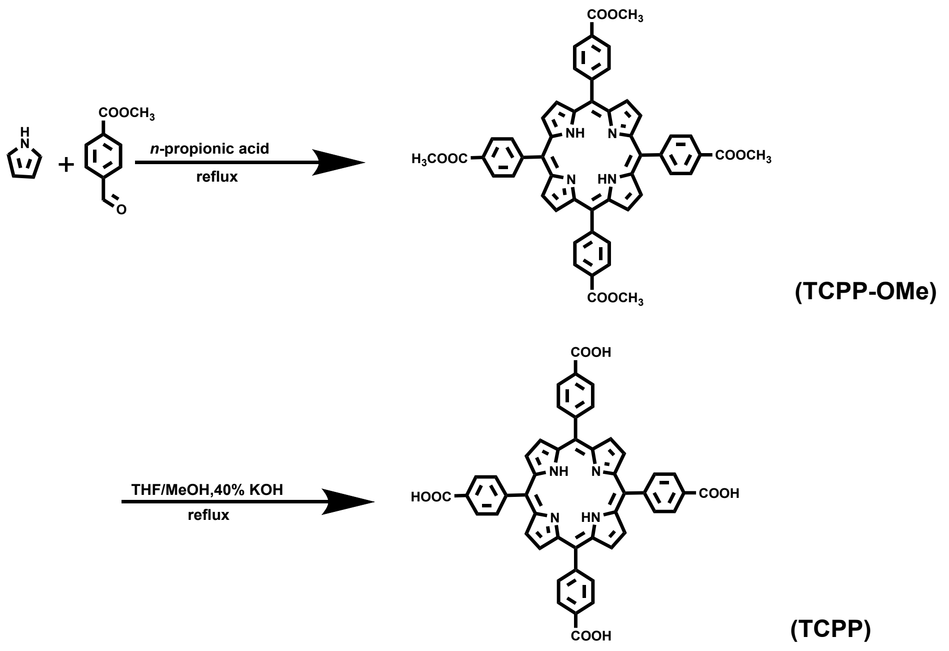

Scheme S1 synthetic procedure for TCPP

1.2 ^{1}H NMR spectra.

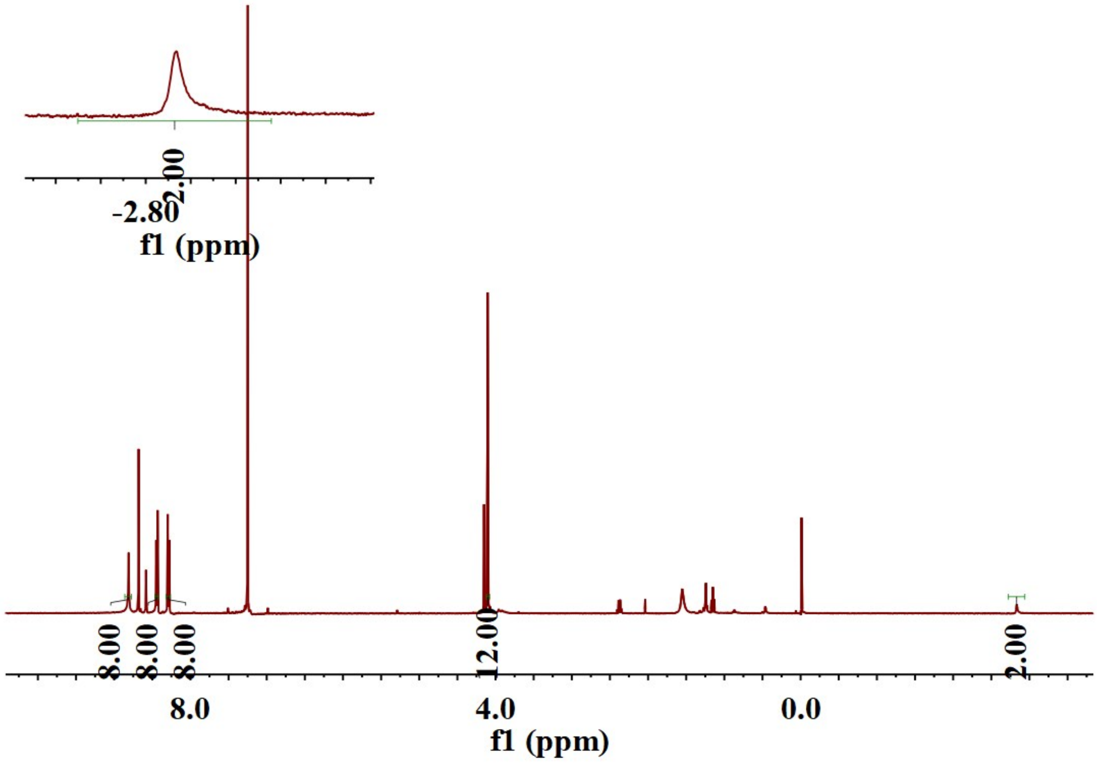

Fig. S1 ^{1}H-NMR spectrum of TCPP-OMe

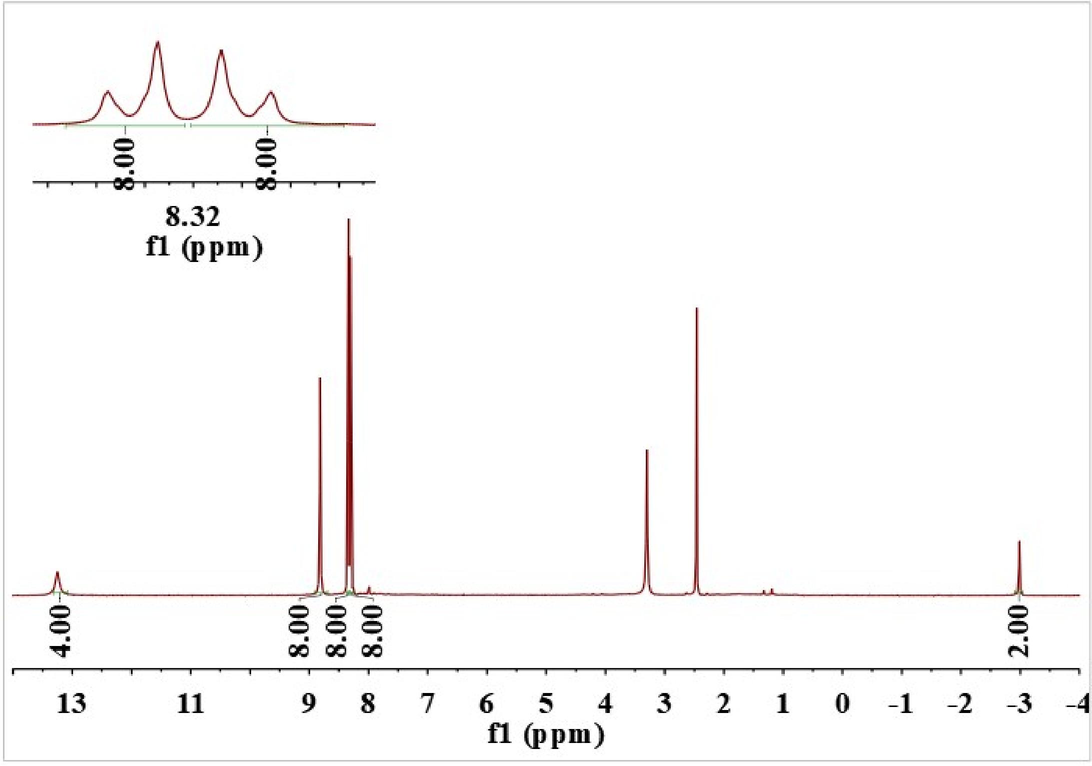

Fig. S2 ^{1}H-NMR spectrum of TCPP

2. Results and discussion

2.1 TEM images of PCNH.

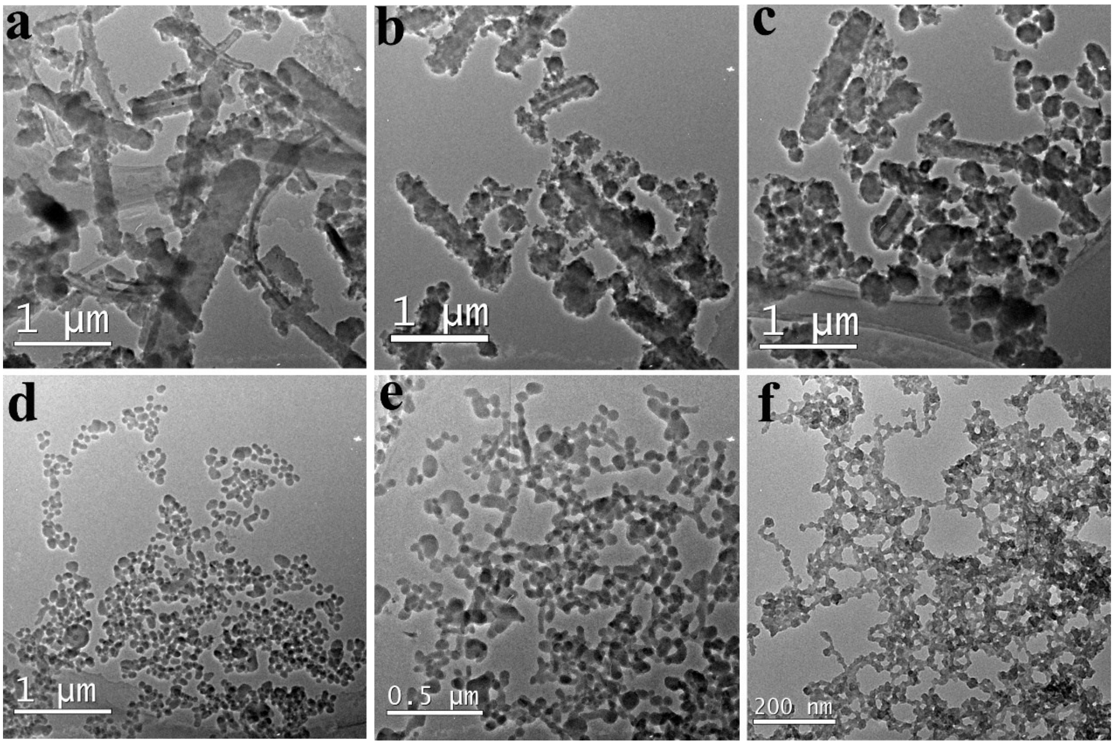

Fig. S3 TEM image of PCNH with 80 μL (a),120 μL(b),160 μL (c), 320 μL (d), 640 μL(e) and in water (pH = 8).

2.2 ξ potential of the samples

	TCPP	cis-platinum	PCNH
ζ potential (mV)	**-14.7**	**+1.1**	**-8.7**

Table S1 ξ potential of TCPP, cis - platinum and PCNH.

2.3 X-Ray photoelectron spectroscopy (XPS)

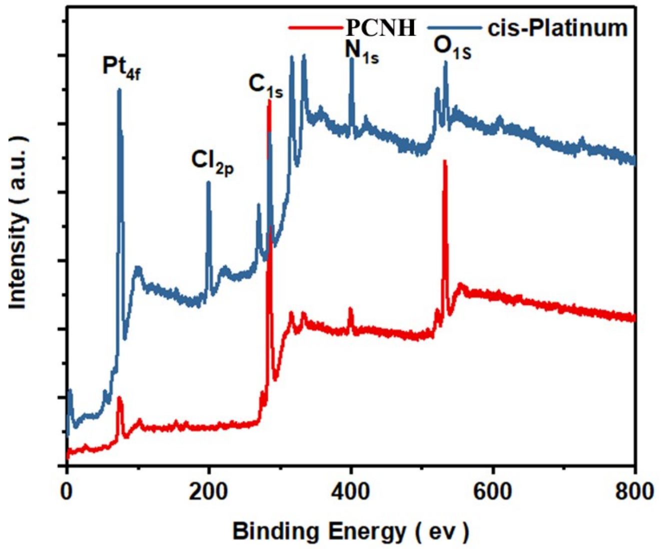

Fig. S4 XPS of cis-Platinum and PCNH

2.4 Fluorescence spectra of singlet oxygen

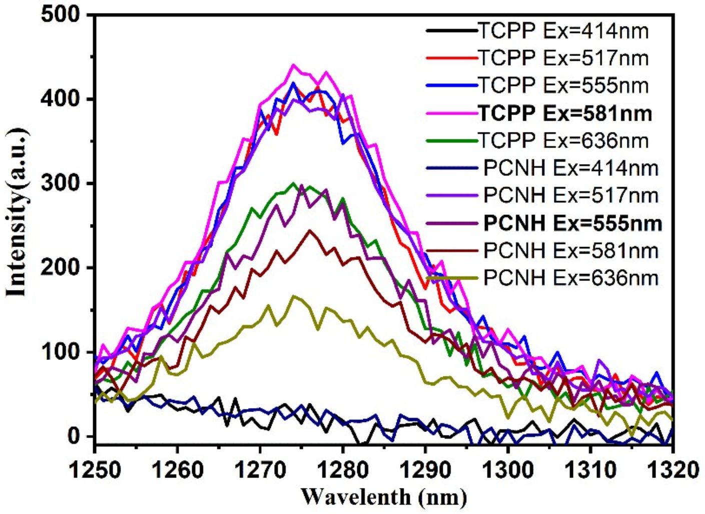

Fig. S5 Fluorescence spectra of singlet oxygen of TCPP and PCNH excited by light with different wavelength.

2.5 UV-vis absorbance spectrum of TCPP and PCNH

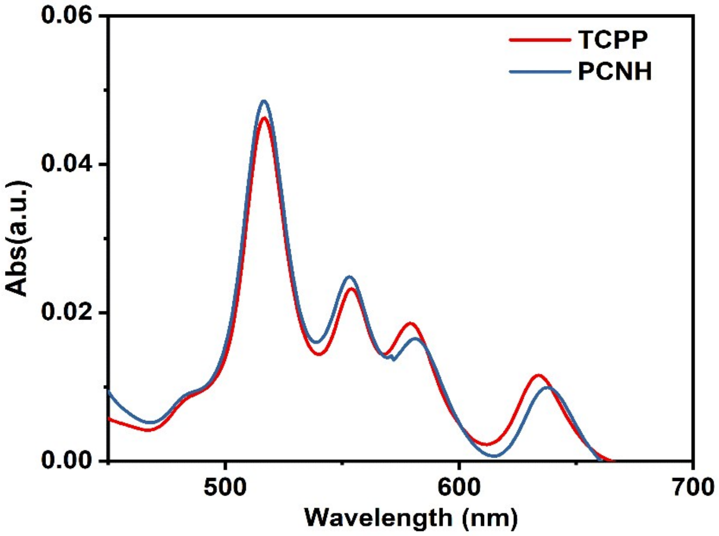

Fig. S6 UV-Vis spectroscopy of TCPP and PCNH.

2.6 Different photostability between TCPP and PCNH

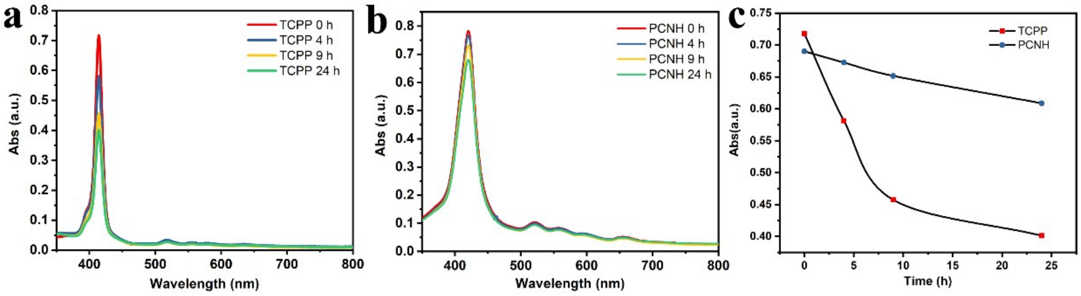

Fig. S7 Absorption spectrum of TCPP (a) ,PCNH (b) and downtrend (419 nm) at special time.

2.7 Cell viability against different concentration of TCPP, cis-platinum and PCNH

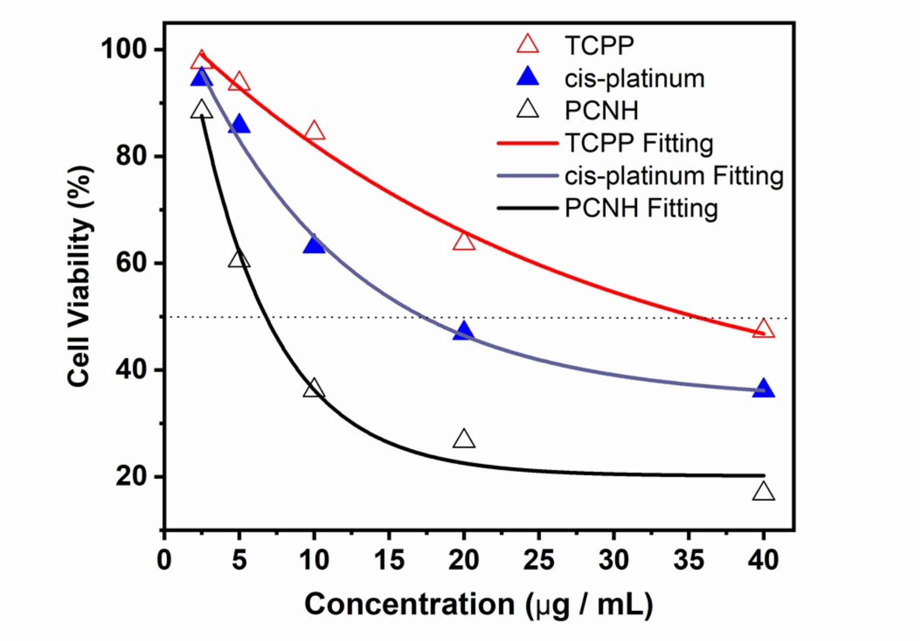

Fig. S8 Plot of cell viability against different concentration of TCPP, cis - platinum and PCNH. (dash line corresponds to IC_{50}).
